# Tissue Penetration of Antimicrobials in Intensive Care Unit Patients: A Systematic Review—Part I

**DOI:** 10.3390/antibiotics11091164

**Published:** 2022-08-29

**Authors:** Stefano Finazzi, Giacomo Luci, Carlo Olivieri, Martin Langer, Giulia Mandelli, Alberto Corona, Bruno Viaggi, Antonello Di Paolo

**Affiliations:** 1 Istituto di Ricerche Farmacologiche Mario Negri IRCCS, 24020 Ranica, Italy; 2 Associazione GiViTI, c/o Istituto di Ricerche Farmacologiche Mario Negri IRCCS, 20156 Milan, Italy; 3 Department of Clinical and Experimental Medicine, University of Pisa, 56126 Pisa, Italy; 4 Anesthesia and Intensive Care, Sant’Andrea Hospital, ASL VC, 13100 Vercelli, Italy; 5 ICU and Accident & Emergency Department, ASST Valcamonica, 25043 Breno, Italy; 6 Department of Anesthesiology, Neuro-Intensive Care Unit, Florence Careggi University Hospital, 50139 Florence, Italy

**Keywords:** antibacterial, penetration, critically ill patient, intensive care unit, beta-lactams, glycopeptides, daptomycin, fosfomycin

## Abstract

The challenging severity of some infections, especially in critically ill patients, makes the diffusion of antimicrobial drugs within tissues one of the cornerstones of chemotherapy. The knowledge of how antibacterial agents penetrate tissues may come from different sources: preclinical studies in animal models, phase I–III clinical trials and post-registration studies. However, the particular physiopathology of critically ill patients may significantly alter drug pharmacokinetics. Indeed, changes in interstitial volumes (the *third space*) and/or in glomerular filtration ratio may influence the achievement of bactericidal concentrations in peripheral compartments, while inflammation can alter the systemic distribution of some drugs. On the contrary, other antibacterial agents may reach high and effective concentrations thanks to the increased tissue accumulation of macrophages and neutrophils. Therefore, the present review explores the tissue distribution of beta-lactams and other antimicrobials acting on the cell wall and cytoplasmic membrane of bacteria in critically ill patients. A systematic search of articles was performed according to PRISMA guidelines, and tissue/plasma penetration ratios were collected. Results showed a highly variable passage of drugs within tissues, while large interindividual variability may represent a hurdle which must be overcome to achieve therapeutic concentrations in some compartments. To solve that issue, off-label dosing regimens could represent an effective solution in particular conditions.

## 1. Introduction

Infections remain one of the most frequent causes of death in intensive care units (ICUs) due to several concomitant factors that severely affect the survival rate of patients. Moreover, about one-fourth of those infections are acquired during ICU stay [1] and, in particular during the COVID-19 pandemic [2], led to specific guidelines aimed at preventing healthcare-associated infections [3]. The landscape is clearly complicated by the presence of hospital strains with the lowest sensitivity to antimicrobials and characterized by strong resistance. Therefore, there is a need for timely antibiotic therapy [4,5], and several guidelines are available to guide physicians in the prescription of the most effective treatments [6,7]. An additional requirement for effective antibacterial chemotherapy is represented by the adoption of therapeutic drug monitoring (TDM) protocols that may be helpful for further increasing therapy appropriateness [8,9], as stressed by Abdul-Aziz and colleagues in a recent position paper [10].

Rapid physiopathological changes in several systems and organs can occur in ICU patients, and they may significantly alter the pharmacokinetics and the efficacy of antimicrobial drugs [11,12,13]. Furthermore, the *tout court* application of pharmacokinetic/pharmacodynamic (PK/PD) parameters may be difficult, and the prediction of treatment efficacy can be arduous. This issue is especially relevant for patients affected by severe infections or deep infective foci, in whom the correlation between drug concentrations in plasma and tissues may be unpredictable. Infections of the central nervous systems (CNS) are paradigmatic examples, because drug diffusion depends on meningeal permeability according to both the inflammatory status of the barrier and the physicochemical characteristics of the antimicrobials [14].

Several clinical trials have investigated the tissue penetration of antimicrobial drugs, and many findings have been collected in healthy volunteers (HV) and patients. However, based on the previous paragraph, the pathophysiological changes in ICU patients can significantly affect the PK of antimicrobials, resulting in different tissue penetration rates compared to what occurs in HV. Furthermore, a wide variability in pharmacokinetic parameters has been observed among ICU patients, making the *a priori* dose adjustment problematic in the absence of TDM protocols.

Therefore, the present review is aimed at harvesting all of the evidence relating to tissue penetration of antimicrobials acting on the cell wall and bacterial membrane in ICU patients and showing the tissue/plasma ratio (i.e., the penetration ratio) at the doses used. These data may facilitate the prescription of the most appropriate antimicrobial regimens depending on the site of infection.

## 2. Results

**Figure 1:**
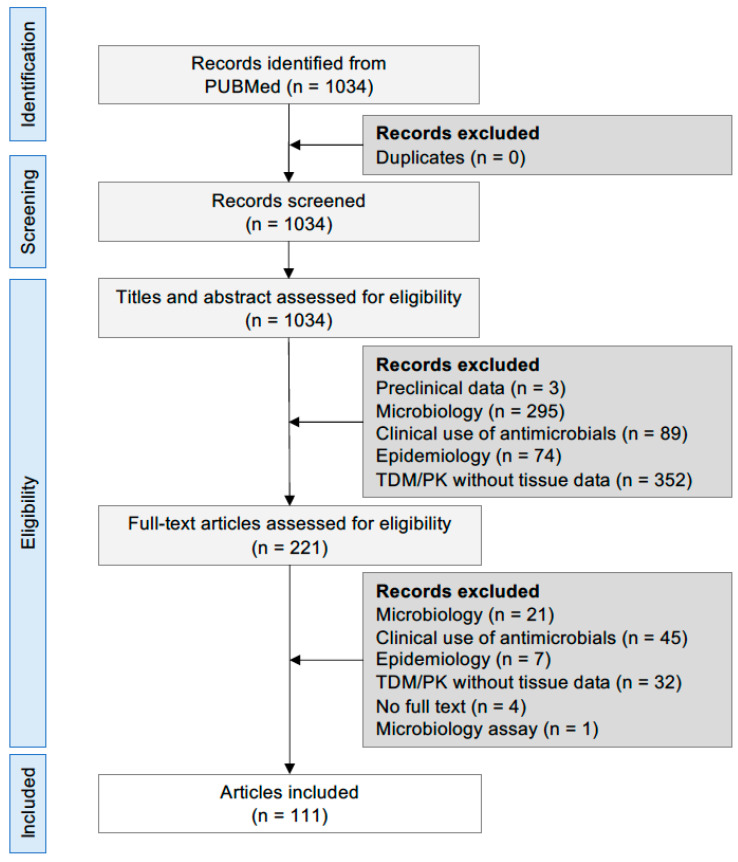
PRISMA flow diagram. Abbreviations: TDM, therapeutic drug monitoring; PK, pharmacokinetics.

The search of articles resulted in 1034 articles, of which 111 remained according to the inclusion/exclusion criteria, as depicted in Figure 1. An additional 16 articles describing tissue penetration in individuals other than ICU patients are included in the text. In the following sections, the tissue penetration of antimicrobials acting on the cell membrane and bacterial wall is described, taking as reference the corresponding plasma concentrations. Additional information regarding the attainment of PK/PD targets at the administered doses is included in the text.

### 2.1. Beta-Lactams

#### 2.1.1. Penicillins

Antimicrobials belonging to the penicillin class are largely used in ICUs to treat both Gram+ and Gram− infections, often in combination with other drugs, because they reach therapeutic concentrations in the respiratory system, abdomen, bone and skin. Furthermore, penicillins, and all beta-lactams, demonstrate time-dependent killing with short or no post-antibiotic effect. The PK/PD parameter predictive of drug efficacy is represented by the percentage time between two consecutive doses during which drug plasma concentrations are above the MIC value (i.e., T > MIC) [15]. The T > MIC value for penicillins is 50%, but, in the presence of resistant strains or difficult-to-treat infections, the efficacy threshold is increased (i.e., T > 4 × MIC).

The diffusion of the drugs within the **CNS** appears to be of paramount importance when treating severe infections. However, the distribution of penicillin into cerebrospinal fluid (**CSF**) is hampered by the hydrophilic nature of the drugs, with a gross CSF/serum ratio value of about 0.1X or less, whereas it is accelerated by meningeal inflammation [14]. As discussed elsewhere, the use of corticosteroids to diminish meningeal inflammation leads to normal permeability that may progressively reduce the diffusion of penicillin through the blood–brain barrier (BBB).

**Table 1:** Tissue/plasma ratio values for some beta-lactams. For beta-lactams/beta-lactamase inhibitor combinations, ratio values are within square brackets. The different daily doses administered to ICU patients are listed in the table.

Drugs	Piperacillin (Pip/Tazo)	Ceftazidime	Ceftriaxone	Cefepime	Meropenem (Mer/Vab)	Imipenem [Imip/Rel]
Daily doses	−4.5 g q6h −22 g CI	−6 g CI −20 mg/kg q8h −2 g q8h	−1–2 g	−2 g q12h −4 g CI	−1 g q8h −2 g q8h EI or CI −[2 + 2 g q8h, 3 h-inf]	−1 g q6h −[0.5/0.25 g q6h]
CNS		0.24X (2 h)	<0.1X (2 h)		0.21X (2.5 h)	
CSF	<0.1X			0.3–2.14X ^A^ 0.03–1.14X ^B^	0.08X	<0.1X (1–4 h)
Lung tissue	0.28 (0.5 h)–0.92X (1.5 h)		0.45X (1 h)	>0.8X (0.5 h)		
ELF	[0.4–0.5X/0.65–0.85X]	0.21–0.44X 0.21X		1X	0.25–0.3X [0.52–1.85X/0.44–0.74X]	0.2X (2 h) [0.3–0.6X/0.3–0.5X]
Bronchial secretion	0.02–0.25X	0.76X	0.02X			
Bronchial mucosa				0.6X		
Abdomen	0.43–0.53X ^C^	0.35–0.56X ^D^	0.09X ^E^	0.51X	0.74X ^D^	
Bone	0.1–0.4X 1X [0.5/0.4X] ^F^	0.1–0.3X (0.5 h)	0.48X (1.5 h) 0.1–0.4X (2–2.5 h)	0.87–1.06X	0.4–1X	0.4X >0.5X
Skin	0.6–0.95X		0.53X (4 h)	>1X (2 h)	>0.8X	
ISF	1X		1X		0.60–0.74X	
Muscle	0.18–0.3X					0.1X
Subcutis	0.1–0.2X				0.44–0.57X	0.14X
Fat	0.1X				0.8X	
References	[16,17,18,19,20,21,22]	[20,23,24]	[25,26,27]	[20,28,29,30,31]	[14,20,24,32,33,34,35,36]	[14,20]

**Notes**: ^A^, ventricular drainage; ^B^, lumbar puncture drainage; ^C^, gut; ^D^, peritoneum; ^E^, abdominal fat; ^F^, synovial fluid. **Abbreviations:** CI, continuous infusion; CNS, central nervous system; CSF, cerebrospinal fluid; EI, extended infusion (i.e., 3 h infusion); ELF, epithelial lining fluid; Imip/Rel, imipenem/relebactam; inf, infusion; ISF, interstitial fluid; Mer/Vab, meropenem/vaborbactam; Pip/Tazo, piperacillin/tazobactam; q6h, q8h and q12h, every 6, 8 and 12 h, respectively.

In ICUs, piperacillin in combination with tazobactam (Pip/Tazo) is the most frequently studied penicillin in relation to its distribution in organs and tissues (Table 1). Many studies addressed the investigation of Pip/Tazo diffusion in the respiratory system of critically ill patients. In particular, a population pharmacokinetic (POP/PK) study in 53 critically ill patients demonstrated that the median ratio between the epithelial lining fluid (ELF) and plasma concentrations of piperacillin was 0.54X after doses of 4/0.5 g every 8 h (q8h) or 6 h (q6h) [21]. These data matched the penetration ratios of 0.57/0.91X for Pip/Tazo 4.5 h after the end of intermittent infusions of 4/0.5 g q8h in 40 patients affected by ventilatory-associated pneumonia (VAP) [37] and 0.4–0.5/0.65–0.85X following continuous infusions of 4/0.5 g q6h in 10 VAP individuals [17]. Similar results were obtained in 17 critically ill patients who received Pip/Tazo 4/0.5 g q8h or every 12 h (q12h) according to renal function [38]. When the area under the time–concentration curve (AUC) values were taken into consideration, the ELF/plasma AUC ratio values of Pip/Tazo were 0.49 (range, 0.02–5.16)/1.21 (range, 0.11–3.91), hence, showing a large interpatient variability. More interestingly, a PK/PD analysis demonstrated that only 54% and 20% of patients achieved T > MIC values of 50% and 100% for MIC ≥ 16 mg/L after being treated with standard regimens, while a minimum plasma concentration (C_min_)/MIC value > 3.4 (which is needed to prevent mutant selection) was predicted in only 6% of patients.

After an i.v. dose of 4/0.5 g Pip/Tazo, the mean tissue/plasma AUC ratio was 0.63/1.93X in the interstitial space fluid (ISF) of the lung parenchyma following microdialysis in five pneumonia patients [39]. Reduced penetration was measured in bronchial secretions, with a ratio ranging from 0.16X to 0.36X [22,40].

Amoxicillin/clavulanate had ELF concentrations of 0.13X/0.18X of the corresponding serum levels, respectively [41]. Higher penetration ratios were measured in bronchial mucosa (i.e., 0.43/0.31X). This study used a microbiological assay to measure tissue and plasma concentrations instead of adopting chromatographic methods.

At a standard dose of 4/0.5 g q8h, Pip/Tazo diffused into the ISF, with ISF/plasma ratio values ranging from 0.2X [42] to 1X, regardless of the type of drug administration (i.e., short or continuous infusions) [18], even in the presence of hemodialytic procedures (i.e., continuous veno-venous hemodiafiltration, CVVHDF) [22]. Indeed, standard doses (4 g q8h) ensured the achievement of T > MIC values for free drug plasma concentrations (*f*T > MIC) equal to 100% [43], while continuous infusions were associated with better results (i.e., 100% *f*T > 4 × MIC). It is worth noting that a tazobactam ISF/plasma ratio of 1X was also demonstrated [19]. These results were paired with those obtained from skin samples from colorectal cancer patients who received a single dose of Pip/Tazo 4/0.5 g before surgery [44], because the mean tissue/plasma ratios were 0.6–1.1/0.5–0.9X in the time interval 0.5–4.5 h after drug administration. It is likely that the majority of patients could attain that tissue distribution after receiving the higher daily doses (i.e., 16/2 g) that are now in use in ICU settings. However, the comparison of the ISF/plasma AUC ratio between patients (range, 0.25–0.27X) and HV (0.43–1.22X) showed that the piperacillin penetration in target tissue was reduced in ICU patients [45]. Finally, ampicillin penetrates into the skin with a tissue/plasma ratio of 0.95X [46].

The penetration of penicillins into the bone varies from 0.1X to 0.4X depending on the drug, but all of the data were obtained in patients other than those admitted to ICUs [47].

Bone/plasma ratios of amoxicillin/clavulanate were 0.18–0.2/0.10–0.15X in 20 patients undergoing orthopedic surgery [48]. Pip/Tazo showed similar penetration into bone tissues with ratios of 0.15/0.13X [49].

#### 2.1.2. Cephalosporins

Cephalosporins are used in ICUs to treat a wide range of infections thanks to their higher rates of penetration in tissues and organs (Table 1). As described above, the PK/PD efficacy index for cephalosporins is the T > MIC parameter. In some studies, the unbound plasma concentration of the drug was considered for the calculation of the PK/PD value (*f*T > MIC), as in the case of ceftazidime [50].

The diffusion of cephalosporins into **CSF** is consistently variable, but, unlike for penicillin, higher CSF/plasma ratios can be calculated for some members of this antimicrobial class. Cefazolin, ceftriaxone and cefixime display a reduced CSF distribution (lower than 0.1X) [14]. A similar penetration rate was measured for ceftarolin (with daily doses of 0.3–0.6 g q12h) in five patients admitted to a neurosurgical ICU [51], while the CSF/serum AUC ratio for cefoperazone was 0.15X [52]. On the contrary, in a French study, which enrolled 16 patients treated with high doses of ceftriaxone (77–131 mg/kg), the CSF/plasma ratio was highly variable (0.01–1.03 X) when CSF samples were collected 1.6–24 h after dosing both by lumbar puncture (LPD) and external ventricular drainage (EVD) [53]. The different sampling route may explain the large variability in CSF concentrations.

In eight patients with occlusive, non-inflammatory hydrocephalus, a single dose of ceftazidime 3 g administered by a short i.v. infusion yielded a median CSF/plasma ratio of 0.05X (range, 0.03–0.12X) [54]. A subsequent study evaluating ceftazidime 2 g described an increased diffusion across the BBB, reaching a maximum CSF/plasma ratio of 0.24X [14]. Cefotaxime 50 mg/kg q8h attained an even greater median CFS/plasma ratio of 0.28X (range, 0.06–0.76X) in 30 newborns and infants (aged 25–47 weeks) [55]. Of note, the blood–brain barrier in neonates and infants displays functions similar to that found in adults, but the age-dependent development of this barrier could explain the variability in drug diffusion across ages [56]. Finally, in eight ICU patients with EVD, cefepime 2 g led to CSF/plasma ratios of 0.30–2.14X even in the absence of meningeal inflammation [30].

Cephalosporins diffuse into the lungs and are used to treat pneumonia. In particular, the passage of cephalosporins into the ELF is comparable to that of penicillin or at least to that of Pip/Tazo, with ELF/plasma ratios greater than 0.2–0.3X. In 15 ICU VAP patients who received ceftazidime (2 g loading dose (LD), 4 g/day maintenance dose (MD) as a continuous infusion (CI)), the mean ELF/plasma ratio was 0.21X [23]. The CI of ceftazidime in VAP patients seemed to have an advantage over intermittent infusion in terms of PK/PD target attainment. Indeed, in the first 48 h of treatment, Cousson and colleagues demonstrated that CI (20 mg/kg LD plus 60 mg/kg/day MD) was associated with ELF concentrations >20 mg/L for a longer time than intermittent infusions (20 mg/kg q8h) [57]. The authors chose a MIC value of 20 mg/L, in agreement with the local ecology of bacterial strains, and that value was similar to the actual epidemiological cut-off (ECOFF) value for resistant strains identified by EUCAST [58].

Interestingly, in 43 HV enrolled in a phase-I study, the ELF/plasma AUC ratio values of ceftazidime/avibactam were 0.31–0.32/0.32–0.35X, with a dose-dependent increase from 2/0.5 g to 3/1 g [59]. The subsequent application of a POP/PK analysis in the same subjects increased the ELF ratios to 0.52X and 0.42X for ceftazidime and avibactam, respectively [60]. Although the examined population included HV, these data deserve to receive interest in relation to the future use of the new beta-lactamase inhibitor avibactam.

Twenty ICU VAP patients received cefepime as a LD (2 g) followed by CI (4 g) for 24 h [31]. The measured ELF/plasma ratio at steady state was approximately equal to 1X, suggesting a high distribution of the fourth-generation cephalosporin.

Of note, ceftolozane diffused into the ELF to a higher degree than piperacillin, as calculated in 26 critically ill patients who required mechanical ventilation [61]. Indeed, the fifth-generation cephalosporin (ceftolozane/tazobactam 3 g q8h as a 1 h i.v. infusion) had a mean ELF/plasma AUC ratio of 0.50X, a value very similar to that formerly calculated in HV (0.48X) [62].

The penetration of cephalosporins into bronchial secretion is extremely variable. In two former studies, ceftazidime penetration into bronchial secretion was found to be in the range 0.12–0.76X for doses of 1–3 g/day [63,64]. On the contrary, a subsequent study tested the presence of five cephalosporins (cefuroxime 70 mg/kg, cefamandole 110 mg/kg, ceftriaxone 80 mg/kg, ceftazidime 80 mg/kg, cefepime 80 mg/kg) in the bronchial secretions and sputum of ICU patients after repetitive administration. Tissue/plasma ratios were approximately 0.02X and 0.05X for cefepime and ceftazidime, respectively, 6 h after the last dose on the second day [26].

Skin penetration values range from 0.38X for cefuroxime and 0.68X for cefixime up to >1X for cefepime. Another cephalosporin, cefazoline (1 g as a single i.v. dose), achieved a median ISF/plasma AUC ratio of 0.74X in 30 ICU patients [65], suggesting high and rapid penetration. Using a microdialysis technique, the subcutis ISF/free plasma ratios of cefuroxime were 0.46X and 0.29X when the drug was administered as a CI (3 g/day preceded by an i.v. bolus of 1.5 g) and i.v. boluses (1.5 g twice a day), respectively [66]. These findings are interesting, because short infusions of ceftazidime in HV resulted in higher AUC values than i.v. boluses, while maximum ISF concentrations were measured 0.5–1 h after drug administration [67]. Moreover, an inverse linear correlation (r = −0.67) between plasma protein binding and ISF/plasma diffusion rate was identified.

Higher ISF/plasma ratio values (1.06X) were attained by cefazolin in seven patients with lower limb infections who received doses of 1 g q8h or 2 g every 24 h (q24h) [68]. A similar ISF penetration rate was demonstrated in 12 patients treated with cefazolin 2 g and who underwent elective or semi-elective surgery for aortic aneurysm [69]. Cefepime 2 g q12h yielded a tissue/plasma ratio of 1.52X (range 0.42–5.06X) in six ICU burn patients [70].

The muscle/unbound plasma ratio at steady state was 0.64–0.82X for cefazolin (mean dose, 178.6 mg/h CI) and 0.23–0.24X for ceftriaxone (mean dose, 330 mg/h CI) [71]. The disposition of ceftarolin (600 mg q8h or q12h) in muscle and the subcutis achieved tissue/unbound plasma AUC ratios of 0.67X and 0.75X, respectively, in 12 male HV at steady state [72]. Interestingly, the q8h regimen was associated with greater AUC and *f*T > MIC values in muscle and the subcutis (50.9% and 52.9%, respectively) compared to the other regimens evaluated (39.0% and 38.2%, respectively). In agreement with these findings, a single dose of ceftobiprole 0.5 g achieved mean tissue/unbound plasma AUC ratios of 0.69X and 0.49X in muscle and adipose tissue, respectively [73], which corresponded to *f*T > MIC values of 40% for MIC ≤ 2 mg/L. Finally, the ISF/plasma ratio was 1.32X in six HV who received a single dose of cefixime 0.4 g [74].

The penetration of cephalosporins into bone has been evaluated at different times after drug administration, and the retrieved data are concordant with a tissue/plasma ratio of ≥0.1 X. In particular, a single dose of cefepime 2 g yielded tissue/plasma ratios >0.8X in both cancellous (1.06 ± 0.23X) and cortical bone tissues (0.87 ± 0.37X) 1.5 h after drug administration [29]. In orthopedic patients who underwent elective orthopedic surgery, ceftriaxone 2 g had lower penetration in both cancellous (0.24X) and cortical bone (0.09X) 1.5–8 h after drug administration [27]. Although the lowest tissue/plasma ratio, the T > MIC value, was 100% for all of the bacterial species causative of the infections (MIC values, 0.25–0.5 mg/L). A further study showed how the tissue/plasma ratio for cancellous bone increased from 0.08X (1 h post dose) to 1.12X 5 h after the administration of ceftriaxone 2 g [75]. Finally, ceftazidime 2 g reached tissue/plasma ratios of 0.03–0.08X in lower limb bone tissues of 10 patients who underwent amputation [76]. The penetration of cefazolin, ceftazidime or ceftriaxone (2 g each) into the nucleus pulposus of 22 patients undergoing elective vertebral surgery was always lower than 0.06X 60 min after drug administration [77].

The distribution of cephalosporins into **abdominal** organs and **peritoneal fluid** (PF) has been described for cefepime, with tissue/plasma ratios of about 0.5–0.7X [28,78]. In agreement with those findings, the peritoneal fluid/plasma ratio of ceftazidime was investigated in 18 patients with intra-abdominal infections treated with intermittent infusions (1.5 g q8h) or CI (1 g as a LD, then 4.5 g/day as CI) [79]. Interestingly, the CI schedule attained higher ratios (0.56–0.64X) than the intermittent infusions (0.33–0.35X).

#### 2.1.3. Carbapenems

In combined regimens, carbapanems, as well as meropenem, imipenem and ertapenem, represent the first-line treatment for severe infections caused by Gram-negative bacterial species. Their use has also expanded to include empirical treatment before antibiogram results are available. Furthermore, the recent emergence of carbapenem-resistant clones (i.e., carbapenemase-producing *K. pneumoniae*) is a challenging situation in ICUs, even though the approval of the meropenem–vaborbactam and imipenem–relebactam combinations has introduced new therapeutic options for the treatment of those severe infections. For these drug combinations, the data regarding tissue penetration are currently very scarce (Table 1). The T > MIC parameter predicts the treatment efficacy of carbapenems [15], and the threshold value is increased in the presence of resistant strains (i.e., T > 4XMIC) [80].

As stated above, the passage of carbapenems across the intact BBB ensures a **CSF** penetration ratio of approximately 0.1X [14]. The inflammation of encephalic barriers permits increased passage (up to 0.2X). CSF samples obtained by EVD better reflect drug concentrations than those obtained by LPD. In 20 patients affected by EVD-associated ventriculitis, the median CSF/serum ratio was 0.09X (range, 0.03–0.16X) [81]. A POP/PK study in 10 ICU patients observed a mean CSF/serum ratio of 0.06X (interquartile range, IQR, 0.02–0.08X) after a short infusion of meropenem 2 g [82,83]. An increased ratio of 0.18X (range, 0.02–0.40X) was obtained in 20 patients who received meropenem according to a 1 g LD followed by a CI of 6 g/day that was subsequently adjusted to achieve CSF concentrations >6 mg/L [84]. Of note, a POP/PK study in newborns found similar results, with a calculated passage of meropenem across the BBB of 0.08X, and CSF penetration depended on proteinorrachia [35]. However, the simulation of prolonged infusions revealed a decreased passage of meropenem within the CSF, because short i.v. infusions may facilitate passive diffusion through increased concentration gradients across the BBB. Another multicenter study in 188 newborns (age ≤ 92 days) found greater penetration of meropenem 20–30 mg/kg q8h or q12h into CSF (0.7X, range, 0.05–0.78X) [85]. As discussed for penicillins, the young age of patients and the progressive development of the BBB across ages could be responsible for the high variability of meropenem passage into CSF.

The disposition of ertapenem 1 g/day in the lower respiratory tract was investigated in VAP patients, showing that the **ELF**/serum ratio was approximately 0.3X (range, 0.12–0.63X) throughout the entire time interval between two consecutive doses [86].

In 39 VAP patients who received meropenem as 30 min (0.5 or 2 g) or 3 h infusions (1 g), the median ELF/plasma AUC ratio was 0.26X, in agreement with a simulated median penetration ratio of 0.26X (90% confidence interval, 0.04–1.78X) [87]. More interestingly, in a study enrolling 55 VAP/healthcare-associated pneumonia (HAP) patients, 3 h infusions of meropenem 1 g yielded greater mean ELF/plasma AUC ratios (0.29X) than short, 30 min infusions (0.20X) [88]. The standard dose of 1 g q8h was ineffective in achieving therapeutic T > MIC values of at least 40–100%, hence, supporting the administration of higher doses (i.e., 2 g as 3 h i.v. infusions q8h). It is worth noting that a recent study in HAP patients demonstrated that, when meropenem was administered as a LD (1 g) followed by CI (1–2 g q8h), the ELF/plasma ratio was 0.32X and 0.36X for total daily doses of 3 g and 6 g, respectively [36]. However, only the highest registered dose of meropenem allowed the attainment of the predefined PK/PD target of 50% *f*T > MIC for MIC values < 4 mg/L. These data strongly support the use of meropenem 2 g q8h as continuous infusions for increasing the probability of an effective treatment, even if the stability of meropenem solutions is limited to 5–8 h [89]. In 48 HV who were treated with meropenem 0.5–2.0 g q8h, mean alveolar cells (AC)/plasma AUC ratios were 0.15X and 0.25X for meropenem doses of 0.5 and 1 g, respectively [90]. For the same dose levels, the mean ELF/plasma AUC ratios were approximately 0.43X and 0.28X.

Meropenem 2 g and vaborbactam 2 g administered as 3 h i.v. infusions had mean/median ELF/unbound plasma ratios of 0.65/0.59X and 0.79/0.72X, respectively, after the third dose [91]. Of note, the tissue/plasma ratio of the beta-lactamase inhibitor in alveolar macrophages (AM) ranged between 0.06X and 2.58X up to 8 h after dosing. These findings suggest that meropenem and vaborbactam (both at doses of 2 g q8h) may achieve effective concentrations in the ELF.

At steady state, the ELF/plasma penetration ratio of imipenem 0.5 g q6h was 0.44X according to the POP/PK model [92]. A subsequent piece of research using HV found that the ELF/plasma and AC/plasma ratios for relebactam 0.25 g were 0.54X and 0.36X, respectively, taking into consideration a plasma protein binding of 20% [93]. Similarly, the ELF/plasma ratio for imipenem was 0.55X (plasma protein binding 20%), but the drug was undetectable in AC. In agreement with the previous study of meropenem/vaborbactam, imipenem/relebactam displayed overlapping concentration profiles in ELF, supporting its use in pneumonia patients. Finally, instillation of imipenem ensured higher concentrations in bronchial secretions than after nebulization [94].

The passage of ertapenem 1 g into **PF** was assessed every 12 h for the first three days of treatment in nine patients with secondary peritonitis [95]. The mean PF/plasma ratio was 1.21X (range, 0.24–2.55X), but the authors concluded that the concentrations were insufficient to adequately treat intra-abdominal infections (IAI), and the daily dose had to be doubled (i.e., 2 g/day). In agreement with those results, meropenem 1 g q8h administered to six patients with peritonitis and septic shock had a PF/plasma ratio of 0.74X, which was considered insufficient to treat infections sustained by intermediately susceptible strains (i.e., MIC = 16 mg/L) [96]. A subsequent simulation of alternative dosing regimens (i.e., meropenem 1 g q6h) showed an improved efficacy (even for MIC = 16 mg/L).

In seven ICU patients (five VAP and two IAI), the tissue/plasma AUC ratio of ertapenem 1 g/day was found to be 0.09X in muscle [97]. In 10 HV who received a single dose of ertapenem 1 g, the tissue/plasma ratios were 0.05X and 0.13X in the subcutis and muscle, respectively [98]. Notably, the ISF concentrations of ertapenem exceeded MIC values for important SSSI pathogens for 7 h and 10 h in the subcutis and muscle, respectively. Increased passage of ertapenem into blister fluid (0.61X) was demonstrated in 12 HV who received 1 g/day for three days [99]. The large variability in tissue penetration could be related to the different techniques used to obtain ISF (i.e., from blister by suction or microdialysis).

In ICU patients requiring CVVHDF (exchange rate, 2–3 L/h), the median ISF/plasma ratio of meropenem 0.5 g q8h was 0.63–0.69X (IQR, 0.6–0.74X) [33]. The PK/PD analysis further showed that a 40% T > MIC was attained in the ISF for MIC ≤ 8 mg/L, but only MIC values ≤ 2 mg/L were associated with a 90% T > MIC in both plasma and the subcutis. These unsatisfactory results were likely due to the low dose of meropenem adopted instead of the higher dosage regimens recommended in critically ill patients (i.e., 2 g q8h). In 10 ICU patients who required extracorporeal membrane oxygenation, meropenem 1–2 g q8h administered as a short infusion achieved a median ISF/unbound plasma ratio of 0.79X [34]. Prolonged or continuous infusions of meropenem 3–6 g/day may significantly increase the probability of PK/PD target attainment (i.e., 100% fT > MIC or 100% fT > 4xMIC) for MIC values equal to 8–16 mg/L.

### 2.2. Glycopeptides

Glycopeptides teicoplanin and vancomycin are hydrophilic drugs, and that characteristic may generally hamper their tissue penetration. The area under the curve (AUC)/MIC ratio can predict the efficacy of vancomycin and teicoplanin (threshold values, ≥400 and >900, respectively), as well as the surrogate marker C_min_ (threshold value 15–20 for MIC = 1 mg/L and 10–30 mg/L, respectively) [100,101,102].

With special reference to teicoplanin, the drug was not detectable in the **CSF** after i.v. doses of 0.4 g q24h [103] (Table 2). It is worth noting that meningitis may significantly influence the penetration of glycopeptides into CSF. Indeed, in 13 ICU patients, the administration of vancomycin (15 mg/kg LD followed by a 50–60 mg/kg/day CI) resulted in greater CFS/plasma ratios in patients with meningitis than in other patients (range, 0.29–0.48X vs. 0.14–0.18X, respectively) [104]. However, the different route of CSF sampling may explain the discrepancies between the CSF/plasma ratios of patient groups. Indeed, CSF samples were obtained by LPD in patients with meningitis and by EVD in the other individuals. The CSF/serum AUC ratio of vancomycin was 0.13X in 16 neurosurgical patients with EVD [105], in agreement with the above results and with a case series of four out of six patients with single time point evaluation [106]. During the steady state of vancomycin (15 mg/kg LD followed by a 60 mg/kg/day CI), the median CSF/plasma ratio in 14 patients was 0.24X (range, 0.18–0.63X) when CSF was collected by LPD [107]. Of note, vancomycin concentrations in CSF were linearly correlated with those measured in plasma. In 29 patients with EVD, the mean CSF/plasma ratio was 0.13X (IQR, 0.07–0.24X) and 0.08X (IQR, 0.05–0.12X) in the bolus and continuous infusion groups, respectively [108]. In both groups, tissue penetration was influenced by the meningeal inflammation. Furthermore, a higher mean CSF/plasma ratio (0.19X) was calculated in 33 patients with EVD who received vancomycin 60 mg/kg/day as a CI [109]. Overall, these findings suggest that a large interindividual variability may characterize the glycopeptide penetration into the CSF of ICU patients. Finally, newborns showed CSF/plasma penetration ratios of 0.26–0.68X [110].

Teicoplanin is usually administered in 3–4 LDs q12h, then treatment continues with daily MDs. When this regimen was adopted in 13 VAP patients, the median penetration rate of teicoplanin 12 mg/kg into the ELF was 1.36X (range, 0.48–3.32X), as calculated at steady state by measuring the ELF and free serum concentrations 18–24 h after the MD [111]. A POP/PK study in 10 HV showed that the median ELF/plasma AUC ratio of vancomycin 1 g after a single dose was 0.68X (range, 0.24–4.33X) [116]. The study further confirmed that a dosing schedule of 1 g q12h was inadequate to achieve desired AUC/MIC values (i.e., ≥400) for MIC > 1. Remarkably, a former study found that vancomycin and albumin concentrations within the ELF of 19 critically ill patients were significantly associated, with albumin being a sign of the inflammatory process in the lower respiratory tract (LRT) [117]. Therefore, when compared with HV, pneumonia patients are characterized by a greater variability of vancomycin concentrations into their ELF depending on the local inflammatory status, and this assumption could be expanded to all hydrophilic drugs. Of note, a simulated regimen of vancomycin 1.5 g q12h (AUC/MIC value ≥ 280) and an ELF/plasma AUC ratio of 0.8X could prevent the emergence of resistant MRSA clones [118].

A single dose of teicoplanin 12 mg/kg in 22 patients undergoing open cardiac surgery achieved measurable concentrations in heart valve tissue up to 10 h after dosing, with a maximum tissue/serum ratio of 0.45X [112]. In the same group of patients, teicoplanin had maximum tissue/serum ratios of 0.49X and 0.57X in muscle and fat, respectively.

In six critically ill patients, the **ISF**/plasma ratios of vancomycin 24 mg/kg/day ranged between 0.47X and 1.34X [115]; consequently, the highest safe dose could be administered to maximize the tissue penetration of the drug. Indeed, in orthopedic patients, a single dose of vancomycin 1 g resulted in median (95% confidence interval) tissue/unbound plasma ratios of 0.31X (0.16–0.46X) in subcutaneous adipose tissue, 0.45X (0.29–0.62X) in cancellous bone and 0.17X (0.11–0.24X) in cortical bone [119]. Taken together, these data confirmed that vancomycin’s penetration rate in soft tissues and bone approaches a value of 0.5X.

### 2.3. Other Antibacterial Drugs

#### 2.3.1. Daptomycin

Daptomycin is effective against Gram-positive strains, and its rapid bactericidal effect supports its use in severe infections of the skin and soft tissues, endocarditis and bacteriemia. The involvement of several Gram-positive strains in other infections has promptly expanded the use of daptomycin. The drug demonstrates concentration-dependent killing that is predicted by AUC/MIC values ≥ 600 and maximum plasma concentration (C_max_)/MIC ratios > 100 [120].

Daptomycin penetration into **CSF** is negligible [14]. In the presence of meningeal inflammation, daptomycin 10 mg/kg achieved C_max_ and C_min_ CSF/serum ratio values of approximately 0.05X in a patient [121]. More recently, in nine patients with healthcare-associated meningitis, daptomycin 10 mg/kg achieved a CSF/serum AUC ratio <0.01X at steady state [122].

In the **ISF** of soft tissues, the unbound tissue/serum AUC ratio ranged from 0.7X to 0.9X in six diabetic patients and six matched HV who received a single dose of daptomycin 4 mg/kg [123]. In 10 diabetic patients receiving daptomycin 6 mg/kg, unbound tissue/serum AUC ratios at steady state were 1.5X in healthy tissues, 1.1X in inflamed subcutaneous adipose tissue and 1.2X in bone [124]. A lower bone penetration was reported in orthopedic patient candidates for knee or hip surgery who received a single dose of daptomycin 8–10 mg/kg [125]. Indeed, bone concentrations (cancellous or cortical bone) ranged between 1 and 8 mg/L 4–12 h post dosing, while the corresponding plasma concentrations were in the range 10–70 mg/L for a median tissue/plasma ratio <0.1X. Interestingly, the median synovial fluid/plasma ratio was 0.54X in the same group of patients. The plasma protein binding of daptomycin accounts for 80–90% [126], and this could explain the conflicting drug penetration rates into bone tissues.

Finally, tissue penetration in bile and heart valves has been demonstrated [127,128].

#### 2.3.2. Fosfomycin

Fosfomycin has regained interest for its role in the treatment of severe infections sustained by resistant strains (i.e., KPC *K. pneumoniae*) in combination with other drugs [129]. Its efficacy is predicted by both time- and concentration-dependent PK/PD parameters [130].

The penetration of fosfomycin 8 g q8h in **CSF** was approximately 0.18X, and the mean AUC ratio was 0.27X [131]. These results were associated with a high probability of target attainment (i.e., >90%) for MIC values up to 16 mg/L, as suggested by another study of ICU patients who received fosfomycin 5 g q8h [132]. The presence of a meningeal inflammation may increase the CSF penetration of fosfomycin [133].

In bronchial secretion, the tissue/serum ratio was 0.13X 2 h after a single i.v. dose of fosfomycin 4 g [134]. More recently, fosfomycin penetration in lung tissues was investigated using microdialysis [135]. After a single dose of 4 g in eight septic patients, the mean tissue/plasma AUC ratio was 0.63X and 0.53X in healthy and infected lung parenchyma, respectively, with corresponding, simulated T > MIC values of at least 80% for MIC = 16 mg/L. Of note, the authors observed a large interpatient variability, which strengthened the use of higher doses (i.e., 24 g/day) even if they were less tolerable.

In the muscle **ISF** of nine septic patients, a single i.v. dose of fosfomycin 8 g was associated with a mean (median) tissue/plasma AUC ratio of 0.70X (0.71X) [136]. An appropriate dosing regimen (i.e., 8 g q12h) was effective against bacterial strains with MIC values of 32 mg/L. In agreement with these findings, patients with cellulitis (n = 6) or diabetic foot (n = 6) received fosfomycin 0.2 g/kg q8h [137]. The calculated tissue/plasma AUC ratios were 0.62–0.70X and 0.60–0.73X in inflamed and healthy tissues, respectively. In nine diabetic patients, a single i.v. dose of fosfomycin 0.1 g/kg resulted in mean tissue/plasma AUC ratios of 0.43X in bone and 0.76X in subcutaneous adipose tissue. These values were judged effective for the treatment of deep-seated infective foci with bone involvement. Using microdialysis, a study in HV treated with a single dose of fosfomycin 4–8 g obtained similar mean tissue/serum AUC ratios in both muscle (0.48–0.53X) and adipose tissue (0.71–0.74X) [138]. This penetration rate was considered effective against *E. cloacae*, *S. marcescens* and *S. aureus* for MIC values up to 16 mg/L.

Finally, in 12 patients, a single dose of fosfomycin 8 g led to highly variable drug concentrations within the abscess ranging from below the detection limit up to 168 mg/L [139]. In order to increase fosfomycin penetration, the suggested dosing regimen consists of a LD of 10–12 g followed by a MD of 8 g q8h in patients with conserved renal function.

#### 2.3.3. Colistin

In recent years, the emergence of multiresistant bacterial clones has brought back colistin (and its prodrug colistin methanesulfonate, CMS) to the clinic for the treatment of severe Gram infections [140]. Of note, several studies in ICU patients confirmed the wide inter- and intrapatient pharmacokinetic variability of colistin [141]. The antibacterial activity of the drug is predicted by the ratio between the AUC value of unbound plasma concentrations and MIC (*f*AUC/MIC) [142].

The CSF penetration rate of colistin is <0.1X [143]; hence, the drug is administered by the intraventricular route, providing better efficacy against resistant strains such as *A. baumanni* [143].

CMS was administered to 13 VAP patients at i.v. doses of 2 million international units (MIU) q8h [144], but, at steady state, the drug was not detected in bronchoalveolar lavage (**BAL**) 2 h after dosing. On the contrary, a case report described an ELF penetration of >7X after administration of CMS 2.8 MIU [20]. Similar results were obtained in two patients treated with CMS 0.225 g q8h, because the BAL/serum ratio was in the range 1.70–7.42X 1.5–4.0 h after the last dose [145]. Even in 12 ICU patients treated with inhaled colistin followed by i.v. CMS, ELF/plasma ratio was >1 for both CMS and colistin at steady state [146]. For these reasons, the variable penetration of colistin within the ELF after i.v. administration supports the inhalation route of high doses for effective treatment of LRT infections caused by Gram-negative bacteria [146]. In addition, the inhalation route may reduce the risk of toxicities because of diminished systemic exposure to the drug.

## 3. Discussion

The issue of the tissue distribution of antimicrobial drugs is of paramount importance in ICU patients due to sickness severity, individual health status and the need to prevent the emergence of drug resistance. Therefore, the penetration rate of a drug within tissues may contribute to the choice of the most appropriate and effective treatments. As reported in the harvested references, there is huge variability in terms of drug penetration among different tissues and organs. Moreover, one major factor of variability is represented by the clinical conditions of severely ill patients. Indeed, drug pharmacokinetics can be influenced by renal failure and the formation of a third space that increases the volume of distribution, sepsis and inflammation. All of these factors may result in a decreased penetration of antimicrobials within infective foci.

Diffusion into the CSF and the brain is challenging for most of the drugs evaluated in the present review. Indeed, beta-lactams and lipo- and glycopeptides achieve concentrations that are only a part of those measured in plasma (i.e., CSF/plasma ratio, <0.1X), and diffusion is slow, with a hysteresis of hours [14]. On the contrary, fosfomycin seems to be able to achieve bactericidal concentration in CSF (for MIC values ≤ 16 mg/L) [131].

Antimicrobials encounter variable penetration into the bone, with tissue/plasma ratios being in the range 0.1–0.4X. Ceftriaxone and fosfomycin attain the highest bone/plasma ratios (approximately 0.4X or more), with PK/PD parameters that suggest antibacterial activity, at least against the most sensitive bacterial strains.

Antimicrobial penetration in the lungs has been demonstrated by beta-lactams and glycopeptides. Indeed, Pip/Tazo achieves ELF and ISF/plasma ratios ≥0.5X [21], meaning that standard dose regimens (i.e., 4/0.5 g q6h) may be considered effective for treatment of infections, but not for preventing the selection of mutant clones in some cases [38]. The administration of beta-lactams by CI, instead of standard short infusions, ensures increased tissue penetration in the ELF [17,36], which, in turn, allows the achievement of better PK/PD goals [57]. Interestingly, the adoption of alternative administration routes has been proven successful for colistin, reaching highest ELF concentrations without exposing the patients to an increased risk of systemic toxicities [146]. Overall, the penetration of beta-lactams within the LRT recommends their use to treat HAP and VAP [147].

Despite the scarcity of data, new beta-lactamase inhibitors can diffuse into the ELF [92,93], and this feature is important for their use in combination with beta-lactams [148].

Another important compartment of drug penetration is skin and muscle ISF. Among beta-lactams, Pip/Tazo, ampicillin and many cephalosporins diffuse into ISF. In particular, clinical trials have demonstrated that the penetration rate of cephalosporins is inversely correlated with plasma protein binding [67]. Notably, data concerning the tissue penetration of antibacterial drugs in burn patients are scarce [70].

The evaluation of the tissue penetration of antimicrobials is characterized by some issues that need to be discussed. In general, one major problem is the lack of common PK endpoints to compare tissue penetration rates across studies. For example, many clinical trials compare drug concentrations in tissues and plasma at the same time after dosing. However, this approach does not consider the delay which occurs before drugs diffuse from plasma to tissues, the so-called hysteresis phenomenon, which is clearly described for the plasma-to-CSF diffusion of antimicrobials [14]. This phenomenon is responsible for the time dependency of the tissue/plasma ratio, because the ratio is low early after dosing, then it gradually increases due to the more rapid clearance of the drug in plasma than in tissues. For that reason, the penetration ratio is based on AUC values for both plasma and tissues, instead of a single sampling time point after dosing. This approach is not always feasible due to ethical concerns and technical difficulties relating to obtaining repetitive tissue samples in the same individuals. In order to overcome those hurdles, the distribution of patients in different groups across the entire sampling interval has been successfully applied for ELF and CSF penetrations together with POP/PK modeling. In other clinical trials, the adoption of microdialysis techniques was helpful.

Furthermore, the evaluation of PK/PD parameters could be problematic for some drugs that demonstrate concentration-dependent killing. Indeed, the calculation of AUC/MIC values may be complicated by the need for several blood withdrawals (as discussed above), while C_max_/MIC evaluation requires the correct identification of time-to-peak time points for tissue sampling in peripheral compartments. In addition, the specific interventions required to exploit the therapeutic benefit of antimicrobials by PK/PD targets remain to be fully elucidated [12,100].

Finally, the present review has some limits. First of all, the collected clinical studies were heterogeneous in terms of the number of patients, type of infections, drug doses and schedules of administration. Therefore, summarizing research findings was difficult. Additional factors of bias included the different time points for blood and tissue sampling, which made the evaluation of PK/PD parameters complicated. Even the methods used to measure drug concentrations in central and peripheral compartments could be considered a source of bias, because former studies mainly employed microbiological assays instead of chromatographic methods. To overcome these limits, the present project performed a literature search according to PRISMA guidelines and adopted stringent criteria for inclusion/exclusion. This approach guaranteed the collection of the most reliable data even if a formal quality assessment of studies [149] was not performed.

In conclusion, standard doses of antimicrobial drugs may not attain therapeutic concentrations in infected tissues, and the studies confirmed the presence of a wide variability in tissue distribution among patients. Obstacles related to the dense collection of tissue samples hinder the evaluation of tissue penetration. However, TDM protocols and alternative administration regimens may help, at least in part, in ameliorating the tissue penetration of antimicrobials in ICU patients. Finally, further studies are needed to improve our knowledge in relation to drug prescription and dosing in severely ill patients.

## 4. Materials and Methods

The present review was based on the following research question: what is known about the tissue penetration of antibacterial drugs in critically ill patients, and, consequently, are standard (and off-label) doses associated with therapeutic tissue concentrations in critically ill patients. The relevance of such a question depends on the penetration rate of antimicrobials in tissues, for which the identified tissue/plasma ratio can be helpful for predicting the clinical efficacy of the standard or off-label chemotherapeutic regimens described in literature.

For the aims of the present review, the selection of drugs was limited to those pharmacological agents acting on the cell wall or cell membrane. Therefore, physicians in ICUs with specialization in infectious diseases and anesthesiology selected a panel of 30 antimicrobial drugs that are commonly used to treat bacterial infections in critically ill patients. The following paragraphs describe the review protocol.

### PRISMA Selection of Literature

Primary original research articles published in peer-reviewed journals were collected from the PubMed database between March and June 2022 by using all of the following keywords organized in 4 main domains:

1. Domain 1: patients and ward: critically ill patient(s) OR intensive care unit OR ICU;
2. Domain 2: study type: (study OR trial) AND (clinical OR human OR case series OR case report);
3. Domain 3: drug list: antimicrobial(s) AND (amoxicillin OR ampicillin OR ampicillin/sulbactam OR aztreonam OR benzylpenicillin OR cefazolin OR cefepime OR cefixime OR cefotaxime OR ceftaroline fosamil OR ceftazidime/avibactam OR ceftazidime OR ceftobiprole OR ceftolozane/tazobactam OR ceftriaxone OR cefuroxime OR daptomycin OR ertapenem OR fosfomycin OR imipenem/cilastatin OR imipenem/cilastatin relebactam OR meropenem OR meropenem/vaborbactam OR oxacillin OR piperacillin OR piperacillin/tazobactam OR teicoplanin OR vancomycin);
4. Domain 4: tissue distribution: tissue AND (distribution OR penetration OR diffusion OR pharmacokinetic(s)) AND (brain OR cerebrospinal fluid OR (epithelial lining fluid OR ELF) OR lung OR bronchial secretion OR skin OR interstitial fluid OR abdomen OR (peritoneal OR peritoneum) OR urine OR kidney OR liver OR bile OR bone OR synovial OR spleen OR muscle OR (subcutaneous OR subcutis) OR fat OR adipose).

The 4 domains were combined by the AND operator, and only full articles in English were retrieved during the first round of the literature search. After this, the second round of the literature search was performed based on the reference list of retrieved articles, and a more specific search was carried out by combining domains 1–3 and distribution in single tissues, organs and compartments (i.e., interstitial fluid, brain, ELF). Search results were managed by Mendeley Desktop software [150] according to the following steps. Duplicates were removed from the database, then two reviewers (G.L. and A.D.P.) selected articles of interest in an independent manner according to PRISMA 2020 guidelines [151], and the selection was performed based on title and abstract if it was informative enough. In particular, all of the articles that were considered suitable for inclusion in the manuscript had to report the following information: the number of patients and the type of infection, drug dose, route and infusion duration (i.e., bolus, extended or CI), frequency of dosing, body fluid and tissue sampling time points and methods for measurement of drug concentrations (i.e., chromatographic methods or microbiological assays). Moreover, further relevant information, as well as the presence of renal replacement therapies (i.e., CVVHDF) and extracorporeal membrane oxygenation, were annotated. Those retrieved papers referring to the following areas of research were excluded: preclinical studies, epidemiology, microbiology, laboratory techniques, clinical use of antibiotics in ICU without explicit reference to tissue penetration of drugs. Additional exclusion criteria were population pharmacokinetics and therapeutic drug monitoring studies if data about drug tissue concentrations were lacking. In the case of controversies, a third reviewer was invited to judge the inclusion of the reference.

All results were expressed as fraction of drug penetration into tissues with respect to the corresponding plasma concentrations. When available, the tissue/plasma ratio (or tissue/free plasma ratio) was preferentially based on relevant pharmacokinetic parameters (i.e., AUC) to exclude possible errors due to the hysteresis by which drugs diffuse from plasma to tissues. When the AUC values were not available, single concentration values were considered (i.e., C_max_), and the time of sampling (i.e., 4 h post dosing) was clearly indicated. The therapeutic regimens (i.e., dose, time interval between consecutive doses and route of administration) were always shown.

## Figures and Tables

**Table 2:** Tissue penetration of glycopeptides.

Drugs	Teicoplanin	Vancomycin
Daily dose	− LD 0.8 g q12h, MD 0.8 g ^A^	− LD 1 g, MD 2 g CI − 1 g q12h − 10 mg/kg q8h
CSF		0.1X (0.16–0.63X) ^B^
Lung tissue		0.4X
ELF	1.46X	0.2X
Bone	0.23X (0.5 h) ^C^	0.13X (1 h), 0.4–0.57X(3 h)
Skin	0.63X (2 h)	
ISF		0.37X
Myocardium	0.25X (valves)	
Muscle	0.25X	
**References**	[111,112]	[24,104,105,107,108,113,114,115]

**Notes**: ^A^, the scheme consists of 3 loading doses (LD) followed by daily maintenance doses (MD); ^B^, range; ^C^, sampling time after dosing. **Abbreviations:** CI, continuous infusion; CSF, cerebrospinal fluid; ELF, epithelial lining fluid; ISF, interstitial fluid; LD, loading dose; MD, maintenance dose; q8h, every 8 h; q12h, every 12 h.

## Data Availability

Not applicable.
